# Co-design of a model for learning conversations about ongoing patient care between medical supervisors and trainees in the rural generalist settings: A research protocol

**DOI:** 10.1371/journal.pone.0351669

**Published:** 2026-06-15

**Authors:** Linda Furness, Janani Pinidiyapathirage, Matthew French, James Ware, Liam Weber, Brendan Carrigan

**Affiliations:** 1 Griffith University, Rural Clinical School, Toowoomba, Queensland, Australia; 2 Rural Medical Education Australia, Toowoomba, Queensland, Australia; 3 Darling Downs Health, Warwick, Queensland, Australia; 4 Darling Downs Health, Kingaroy, Queensland, Australia; University of Science and Technology of Fujairah, YEMEN

## Abstract

**Introduction:**

In rural generalist clinical settings, medical trainees routinely assess patients and discuss their findings with supervisors to plan ongoing care. These interactions termed ‘learning conversations’, serve as important opportunities for workplace-based learning and clinical decision making. However, preliminary evidence and stakeholder feedback indicate a lack of shared understanding regarding how these conversations should be structured, facilitated, and optimised. This study aims to observe current practices, identify supervisor and trainee learning needs, and co-design a model of learning conversations that enhances educational value and supports safe, effective patient care.

**Methods and analysis:**

This study will use an exploratory sequential mixed methods design structured around the 3Cs of co-design (C*o-define*, C*o-design, Co-refine).* Phase 1 (co-define) will involve qualitative observation and audio-recording of learning conversations across two rural hospitals to characterise current practice and inform a protype model. In Phase 2 (co-design), supervisor and trainee focus groups will explore perceived needs, expectations, and feedback on the prototype model. A national online survey of rural supervisors and trainees will further inform model refinement. Phase 3 (co-refine) will incorporate national stakeholder input through a workshop, followed by feasibility testing of the refined model during a pilot simulation at a Rural Clinical School. Outcome data will focus on model usability, perceived relevance, applicability across contexts, and users’ experiences of employing the model during simulated learning conversations.

**Discussion:**

This study will produce a stakeholder-informed model that responds to the specific learning and clinical needs of rural generalist practice. By embedding co-design throughout the research process, the resulting model is expected to strengthen learning conversations, optimise trainee learning, and enhance the quality and safety of patient care. The findings have potential applicability across broader health professional training contexts and can support workforce development in rural healthcare settings.

## Introduction

Workplace learning affords medical trainees pedagogically rich learning opportunities that support the development of their skills in communication and patient care [1]. In the clinical environment medical trainees frequently conduct initial assessments or review patients, before discussing them with supervisors to plan ongoing care. These conversations can be considered learning conversations and serve as important opportunities for workplace-based learning and clinical decision making [2]. In contrast to traditional case discussion or handover which are limited to single episodes, these conversations are iterative, involving multiple phases of a patients care journey and the trainee retains varying levels of responsibility and autonomy. Anecdotally, a mismatch in expectations between supervisors and trainees about how these conversations are conducted and what frameworks are used has been described by medical supervisors engaged in a rural medical training. This highlights the need to co-design a model of learning conversations that would be tailored to clinical context and supervisor and trainee needs.

Effective clinical communication is integral to all aspects of patient care [3], when ineffective there is potential risk for compromised patient safety [3–5]. Learning conversations provide an important platform for effective clinical communication between medical supervisors and trainees regarding ongoing patient care [6]. As they can occur over a wide range of clinical contexts, supervisors and trainees report use of a variety of frameworks [7]. These include frameworks for handover such as ISBAR [8], as well as frameworks developed to support case-based teaching and learning, including SNAPPS [9], One-minute preceptor (OMP) [10], PQRST [5] and WWW-DOC [11]. While widely adopted, there is no literature which examines how and to what extent these frameworks are being used in learning conversations, the effectiveness of their use and applicability to the rural context [7]. Furthermore, there is no evidence to suggest that these frameworks were developed with stakeholder engagement or evaluation as part of the design process [5,8–11]. Co-design in health professional education is an emerging approach, yet structured methodologies and frameworks are lacking [12].

In rural settings, learning conversations between medical trainees (medical students and junior doctors, including Prevocational Junior Doctors, Principal House Officers, Registrars) and supervisors frequently take place in the Emergency Department where trainees conduct an initial assessment of a patient, then discuss their findings and collaboratively develop a plan for ongoing patient care with their supervisor [13]. Similar learning conversations have been reported by medical supervisors as taking place during Emergency Department ward rounds and inpatient wards where trainees present patients to supervisors and receive guidance about ongoing care [2,14,15]. These conversations not only facilitate patient care but also represent an important medium for learning [16]. Overlooking the role of learning conversations between supervisors and trainees as an important medium for learning risks losing valuable opportunities to augment learning through conversation [16].

While there are multiple models and suggested strategies for supervisors to guide trainee skill development when providing care, currently there is no clear understanding of the educational models for facilitating trainee learning through conversations about ongoing patient care, particularly in rural contexts [7]. Responding to needs identified by rural medical supervisors to develop a shared framework, this study aims to co-design a practical model to guide learning conversations in rural medical workplace learning.

## Materials and Methods

### Study design

An exploratory sequential mixed methods design will be used [17] for this study. We will adopt an interpretivist perspective underpinned by constructivist assumptions, acknowledging that knowledge and meaning are co-constructed through the dynamic engagement and contextual interpretation of both participants and researchers [18].

The methodological orientation of this study draws on the 3Cs framework described by Pearce and Magee [19] who explored how co-creation can be applied in welfare, health and educational contexts. They proposed the 3C Framework – co-define, co-design and co-refine- for applying co-creation and co-design in real world settings [19]. The 3Cs framework is underpinned by Participatory Action Research approaches [19] describing the organic, creative and empowering nature of co-creation while acknowledging the barriers to implementation. Co-define aims to establish a shared understanding of the issue being managed and identify the needs of participant stakeholders. Co-design prioritises problems and generates solutions, while co-refine tests, evaluates and prepares for implementation. The 3Cs framework [19] will be reflexively adapted to suit the rural context and our research aims.

### Study timeline

**Fig 1:**
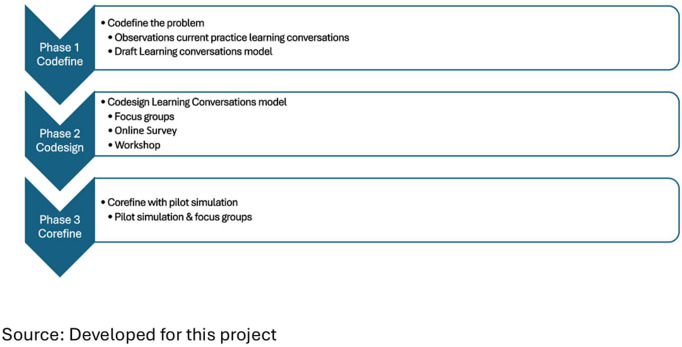
Phases in study.

The study is being conducted over a two-year period in three phases as illustrated in Fig 1- Phases in the study. Phase 1 (co-define), involving observational data collection and development of a prototype learning conversations model, has now been completed and findings from this phase of the study will be reported separately. The prototype learning conversations model developed, reflects the elements of the learning conversations observed. Phase 2 (co-design), which includes supervisor and trainee focus groups, a national online survey, and stakeholder workshop to refine the prototype model, is currently underway and is expected to be completed within the next 2–3 months. Phase 3 (co-refine) will involve pilot simulation of the refined model within a training setting, followed by focus groups to explore supervisor and trainee experiences of using the model. This phase is anticipated to completed in the next 3–4-months. ‌‌Recruitment for the study commenced 3/3/2024 and is expected to be finalised 30/6/2026.

Ethics approval for the study has been obtained from the relevant health service and from the universities that place trainees in the participating rural hospitals. Data will be managed according to Human Research Ethics Committee approvals with de-identified data retained on the Griffith University Research Data Storage Platform.

### Study setting and participants

Two rural hospitals within a regional health service in Queensland, Australia, are the proposed setting for observational and focus group data collection (Phases 1 and 2). These sites are characterised by high clinical diversity and patient volume and were selected for their strong focus on medical education [20,21]. Participants will be medical students, junior doctors (Principal House Officers, Registrars, Prevocational Junior Doctors) and supervisors (Senior Medical Officers). Drawing on our prior experience in the rural health sector, where recruiting active clinicians presents challenges, we will adopt a convenience sampling approach to maximise engagement and participation [22].

A national online survey and national workshop (Phase 2) will be used to reach a national audience of medical students, junior doctors and supervisors across Australia. The online survey will be distributed using purposive sampling, while convenience sampling will be used for the national workshop (Phase 2) to enable efficient recruitment within available timeframe and resources, while ensuring representation from the target population of national supervisors and trainees.

The pilot simulation of the model of learning conversations (Phase 3) will take place at a Rural Clinical School involving medical students completing a longitudinal integrated clerkship [22] and their supervisors.

### Data Collection

#### Phase 1: Co-define using observation and recorded learning conversations.

Phase 1 used qualitative observational research [23] to co-define the problem of a lack of shared understanding of learning conversations, explore current practice, and inform the development of a draft prototype model of learning conversations.

A member of the research team not previously known to participants adopted a nonparticipant observer role, shadowing medical supervisors and making audio recordings of conversations between medical supervisors and trainees about ongoing patient care. Information about the study, was provided to all potential participants by members of the research team prior to the study commencement. Learning conversations about ongoing patient care occurred in settings including the Emergency Department after trainees have completed an initial patient assessment, in Emergency Department ward rounds where trainees present patient updates, and during inpatient ward rounds where trainees discuss patient management with medical supervisors. To meet ethical review committee requirements, recordings were made away from the patient bedside in settings such as in the handover room or staff workspace and excluded patients.

All participants provided written informed consent, with a process of ongoing verbal consent used to confirm agreement to participate. Audio recordings were made of both initial conversations where a trainee presented and discussed a patient’s case to a supervisor, and any subsequent follow-up conversations as patient care progressed. Fieldnotes were compiled by the researcher to capture contextual information, participant roles and relevant environmental factors. A copy of the fieldnote template is provided in Supplementary File 1. Data collection was conducted iteratively and continued until no new concepts or insights emerged. Participation was voluntary with participants able to withdraw their data up to the point that data de-identification occurred. The findings from this phase informed development of a prototype learning conversations model that is being further refined in Phase 2.

#### Phase 2: Co-design using supervisor and trainee focus groups, national online survey and workshop.

During Phase 2, medical supervisors and trainees will be invited to share their perspectives about their needs in learning conversations and provide feedback on the prototype model. Data from Phase 1 will be used to design focus groups for rural hospital supervisors and trainees as well as a national online survey. Data collection at both health service and national levels will capture a broad range of perspectives.

### Supervisor and trainee focus groups

Semi-structured focus groups will be conducted with medical supervisors and trainees from selected rural hospitals to elicit their perspectives about current practice, needs, and expectations of learning conversations. Focus group participants will be facilitated to co-design the learning conversations model through viewing the prototype model developed in Phase 1, assessing its alignment with current practice and suggesting revisions. A semi-structured interview guide informed by data from recorded learning conversations (Phase 1) will be developed for the focus groups.

Members of the research team will visit the sites to introduce the study to potential participants. Supervisors and trainees will be provided with information about the study and invited to attend a focus group via email from hospital or medical school administration. All focus group participants will provide written consent using the Participant Information/ Consent Form prior to the focus group. Acknowledging power differentials, separate focus groups will be held for supervisors and trainees to enable them to freely express their opinions. To further minimise risk of bias, an external facilitator not known to participants will facilitate discussion, and non-identifiable reporting will be used. It is anticipated that 4–6 focus groups will be run, aiming for 5–8 participants in each group with data collection will continue until no new themes are emerging. Focus groups will be audio recorded using handheld recorders. Sonix™ (Sonix.ai, San Francisco, USA) software will be used to transcribe recorded data, a researcher will check and de-identify data before uploading it to NVivo® (QSR International, VIC, Australia) for analysis. Following transcription participants will only be identified as ‘supervisor’ or ‘trainee’. Participants will be invited to review a de-identified copy of their focus group transcript to clarify or add any additional comments.

### National survey

A national survey to capture participant perceptions of the proposed model of learning conversations for ongoing patient care will be hosted in Qualtrics. Prior to distribution the survey will be piloted by several medical educators to check clarity and flow of questions. An email invitation to complete the online survey will be distributed by members of the research team through their networks, including universities, health services, and specialty training college networks, with the estimated reach being approximately 7,000 medical supervisors and trainees across Australia [24]. The survey will take approximately 15 minutes to complete and can be completed using a computer or mobile phone. No identifying details will be collected, and all responses will be anonymous. In accordance with the approved ethics protocol, participant consent will be inferred from completion and submission of the survey. Given the focus of the study is rural medical practice, participants who indicate their location as metropolitan will automatically be exited from the survey. All survey responses will be identified using codes such as ‘Supervisor 1’ or ‘Trainee 1’. Quantitative data from Likert-scale items will be analysed using descriptive statistics to characterise overall response patterns and variability, and thematic analysis of free text responses will be used in the co-design process.

### Workshop

The aim of the workshop is to examine the extent to which the prototype learning conversations model addresses the self-perceived needs of a national group of medical supervisors and trainees. All supervisors and trainees attending a national rural medicine conference will have the opportunity to attend a workshop session facilitated by members of the research team. This interactive workshop will invite participants to share their knowledge and experience of rural generalist workplace learning conversations between supervisors and trainees and provide feedback on the prototype model codesigned in phase 2. Following a presentation about the learning conversations model, participants will be invited to join facilitated small group discussions to share their experiences and perspectives on learning conversations in rural generalist clinical practice. At the commencement of the workshop, attendees will be provided with information about the study, informed that group discussions will be recorded, and given the opportunity to leave the session or not participate in group discussion. Consent will be implied by participants’ decision to attend and engage in the workshop. Small groups will be invited to document their discussions on butcher’s paper, after which a spokesperson will summarise the small group’s key discussion points for the wider group. Group conversations will be audio recorded using handheld recorders, transcribed in Sonix™ and checked by a researcher before being uploaded into NVivo for analysis. Following transcription, data will be identified using a code (e.g., Supervisor 1, Trainee 1). The workshop data will be triangulated with survey data to enhance depth and creditability of findings.

### Phase 3: Co-refine using Pilot simulation

In phase 3, a pilot simulation is proposed to further co-refine the learning conversations model by seeking perspectives of medical supervisors and trainees who have trialled its use within a simulated training environment. All medical students and supervisors attending two consecutive student education days run by the Rural Clinical School will be invited to participate in a training session outlining the model of learning conversations for ongoing patient care. A member of the research team not involved in student training and assessment will introduce the study to potential participants and provide copies of the Participant Information Sheet. Written informed consent from medical supervisors and medical students will be obtained prior to participation in pilot simulations and focus groups. Medical students will be assured on the Participant Information Sheet that their participation in this project does not link to assessment tasks or academic outcomes.

At a subsequent training day, all medical students and supervisors will be invited to participate in simulated learning conversations about three patient scenarios. Each scenario will last 15 minutes, and medical students will have learning conversations with three different supervisors. All medical students and supervisors will then be invited to participate in a focus group to explore their experiences of using the model in a simulated setting. Separate focus groups will be held for supervisors and students to ensure participants are able to share their perspectives freely. Focus groups will be facilitated by a member of the research team not involved in providing education and assessment and will last approximately one hour. It is anticipated that 4–6 focus groups will be run, aiming for 5–8 participants in each group.

### Data management

Audio recordings from learning conversations, focus groups and the workshop will be transcribed using Sonix (Sonix.ai, San Francisco, USA) software, checked and de-identified by a researcher before being uploaded to NVivo® (QSR International, VIC, Australia) for analysis. Once recordings are transcribed, data will be de-identified, and participants will only be identified as ‘supervisor’ or ‘trainee’. Survey data will be uploaded to NVivo® (QSR International, VIC, Australia) for analysis.

### Data analysis

Reflexive Thematic Analysis [25] will be used to analyse data from the three phases of the study. Two members of the research team will familiarise themselves with the data (recordings, focus group transcripts, survey data, workshop transcripts and notes), generate initial codes, and meet to discuss findings to create a preliminary code book. The codebook will be applied by all members of the research team to identify, review, and refine themes before generating the final report. Discrepancies will be resolved through team discussion. NVivo® (QSR International, VIC, Australia) software will be used for analysis and mapping themes.

**Fig 2:**
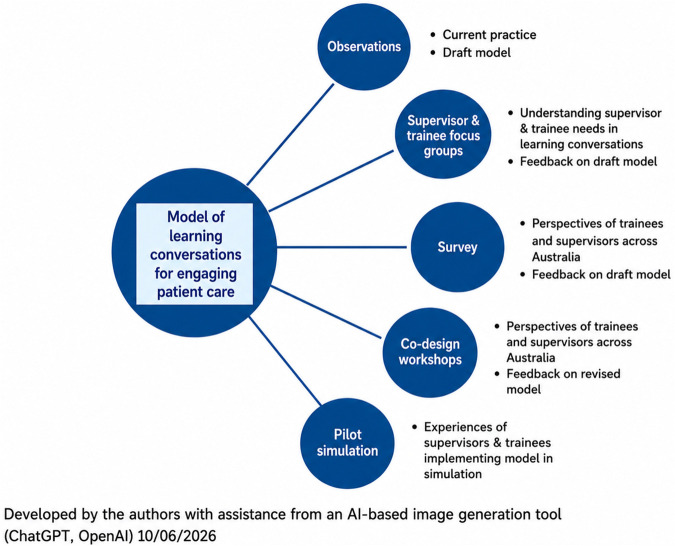
Model development and evaluation.

To enhance the depth and credibility, the study data from each phase of the study will be triangulated [26] and used to co-design a model of learning conversations between medical supervisors and trainees about ongoing patient care as illustrated in Fig 2- Model development and evaluation. While this may limit our ability to guarantee full data saturation, iterative analysis during data collection will be used to monitor emerging themes. Data saturation will be considered to be achieved when themes are repeated, and no new insights are identified across the data.

### Research team and reflexivity

Reflexivity will be considered and discussed throughout the study [27]. The research team bring a range of experiences to the project and acknowledge the assumptions and potential biases which may result from their professional backgrounds, and educational paradigms. The research team comprises rural medical practitioners with expertise in rural practice (BC, MF); clinical medical administrators (JW, LW), an allied health academic (LF), and senior health researcher (JP). This broad experience allows active interpretation and consideration of the data from a range of views and previous experiences. Regular team meetings will be conducted to draw on these unique perspectives to contribute to understanding of the data and learning conversations in practice. Researchers will also actively consider preexisting biases and assumptions to ensure rigor in data analysis [27]. Reflexive practice, including team-based analysis, reflexive journalling and audit trails, will be embedded throughout the study.

## Discussion

The practical application of learning conversations between supervisors and trainees about ongoing patient care is not well understood [7]. Many different frameworks, developed for different contexts are described by supervisors and trainees however the use for learning conversations, particularly in rural contexts, is not clear [7]. It is clear, however that learning conversations are participatory, learning occurs through conversation, with the actions of both supervisors and trainees fundamental to learning outcomes [7,16]. We draw on the expertise and experience of medical supervisors and trainees actively engaged in rural medical workplace learning. Through engaging with research end users at both local and national levels, this protocol outlines a pathway to a co-designed, practical model for learning conversations in a rural context [28].

We present a novel adaption of the 3Cs framework for co-creation [19]. Although originally described in a different context, the 3Cs framework is applicable to co-creation and co-design in real world settings [19]. We propose reflexively adapting the framework to suit the context of learning conversations between medical supervisors and trainees taking place in a rural workplace learning setting. Given funding and participant time restrictions, co-design cannot be applied uniformly across all phases of this study. Instead, it will be intentionally integrated in phases 2 (co-design) and 3 (co-refine) where it can most meaningfully influence the development and refinement of a model of learning conversations. Co-define will focus on observation of participants learning conversations and generation of an initial conceptual model situated in the actual clinical practice of the participants. Through co-design, medical supervisor and trainee’s needs and perspectives will actively adapt the model to further augment workplace learning. Through co-refine, the model will be tested in simulated practice and refined through participant experience and critique. This staged approach emphasises the value of participant engagement to interrogate a preliminary model and ensures participant input enables greatest impact.

The structure of this study maintains methodological rigor, while strategically leveraging the expertise of participants to co-design and refine a model that is fit for use in a rural clinical environment. The resulting practical model will advance teaching and learning in the medical workplace and as a result improve the provision of safe clinical care.

### Strengths

This proposed study applies the 3Cs of co-design; *co-define*, *co-design*, and *co-refine* [19] to develop a model of learning conversation about ongoing care between supervisors and trainees. In contrast to other frameworks supporting case-based teaching and learning such as SNAPPS [9], One-minute preceptor (OMP) [10], PQRST [5] and WWW-DOC [11], it will be developed with stakeholder engagement and evaluation. Validity of the model will be established by piloting the model and gaining supervisor and trainee perspectives throughout the co-refine process.

### Limitations

While this study provides an important contribution to medical education, several limitations should be considered when interpretating the study findings. Due to budgetary and time constraints, recordings of learning conversations and supervisor/trainee focus groups will be conducted in two selected rural hospitals using convenience sampling, which may limit representativeness. The inclusion of a national survey and co-design workshop will provide a broad range of perspectives to increase the generalisability of findings; however, findings should be interpreted with consideration of context.

In response to ethical concerns raised by the Human Research Ethics Committee, patients will not be included in recorded learning conversations, potentially limiting data richness and an understanding of some aspects of these conversations. Future research could include patient perspectives to capture richer contextual data. Whilst trainees may have learning conversations with a range of health professionals (for example, a nurse practitioner in the Emergency Department), only conversations with medical supervisors will be included in this study, which excludes a multiprofessional perspective. Resource and time constraints have necessitated that the pilot testing of the co-designed model be conducted in a simulated setting. Further research is required to evaluate the implementation of the co-designed model in workplace settings, including its feasibility, clinical impact, and applicability within multiprofessional learning environments.

### Potential impact

This study offers insights into the current practice of workplace learning conversations and will co-design a learning conversation model. The model developed will inform workplace learning practices by providing a better understanding of supervisor and trainee perceptions of their needs for learning conversations. The impact of these benefits not only affects future supervisor and student experience and learning, but patients, rural communities and rural health outcomes alike. The model could potentially be used across other health professions whose trainees have learning conversations with supervisors about ongoing patient care.

## Supporting information

S1 File This is the S2 File: Researcher fieldnotes. (DOCX)
