# Supplementary material for: Co-design of a model for learning conversations about ongoing patient care between medical supervisors and trainees in the rural generalist settings: A research protocol
Source: PLoS One. 2026 Jun 15;21(6):e0351669. doi: 10.1371/journal.pone.0351669 (PMC13268152; doi:10.1371/journal.pone.0351669)
Supplement: S1 File — (DOCX) [file pone.0351669.s001.docx]

Supplementary File 1

### Phase 1 Codefine: Researcher Fieldnote template

***Instructions to data collector:***

Please complete this field note for each recorded conversation between medical supervisor and trainee.

***Field note***

- *Medical supervisor:* (use participant number e.g. medical supervisor 1)
- *Medical trainee:* (use participant number e.g. medical trainee 1)
  - Indicate role of medical trainee: Registrar, Junior Doctor, Intern, medical student.
  - If medical student, which university do they attend?
- *Date:*
- *Consent:* verbal consent to be recorded and for recording to be used has been provided by both medical supervisor and medical trainee. Yes/No
- *Location of conversation:*
- *Length of conversation:*
- *Context of conversation:*
  - Please provide any details about the context of the conversation which may help with data analysis. e.g. Conversation related to patient deterioration, discharge planning etc
- *Background:*
  - Note any relevant background information
- *General feel and observations of the conversation:*
- *Reflections on what was said:*
